# Development of machine learning-based clinical decision support system for hepatocellular carcinoma

**DOI:** 10.1038/s41598-020-71796-z

**Published:** 2020-09-09

**Authors:** Gwang Hyeon Choi, Jihye Yun, Jonggi Choi, Danbi Lee, Ju Hyun Shim, Han Chu Lee, Young-Hwa Chung, Yung Sang Lee, Beomhee Park, Namkug Kim, Kang Mo Kim

**Affiliations:** 1 Department of Gastroenterology, Asan Liver Center, Asan Medical Center, University of Ulsan College of Medicine, 88 Olympic-ro 43-gil, Songpa-gu, Seoul, 05505 Korea; 2 Department of Convergence Medicine and Radiology, Asan Medical Center, University of Ulsan College of Medicine, Seoul, Korea

**Keywords:** Medical research, Gastroenterology, Hepatology, Cancer, Gastrointestinal cancer, Computational biology and bioinformatics, Machine learning, Predictive medicine

## Abstract

There is a significant discrepancy between the actual choice for initial treatment option for hepatocellular carcinoma (HCC) and recommendations from the currently used BCLC staging system. We develop a machine learning-based clinical decision support system (CDSS) for recommending initial treatment option in HCC and predicting overall survival (OS). From hospital records of 1,021 consecutive patients with HCC treated at a single centre in Korea between January 2010 and October 2010, we collected information on 61 pretreatment variables, initial treatment, and survival status. Twenty pretreatment key variables were finally selected. We developed the CDSS from the derivation set (*N* = 813) using random forest method and validated it in the validation set (*N* = 208). Among the 1,021 patients (mean age: 56.9 years), 81.8% were male and 77.0% had positive hepatitis B BCLC stages 0, A, B, C, and D were observed in 13.4%, 26.0%, 18.0%, 36.6%, and 6.3% of patients, respectively. The six multi-step classifier model was developed for treatment decision in a hierarchical manner, and showed good performance with 81.0% of accuracy for radiofrequency ablation (RFA) or resection versus not, 88.4% for RFA versus resection, and 76.8% for TACE or not. We also developed seven survival prediction models for each treatment option. Our newly developed HCC-CDSS model showed good performance in terms of treatment recommendation and OS prediction and may be used as a guidance in deciding the initial treatment option for HCC.

## Introduction

Hepatocellular carcinoma (HCC) is the third and seventh most common malignancy in men and women worldwide, respectively, and its incidence continues to increase^1^. The American Association for the Study of Liver Diseases and the European Association for the Study of the Liver currently endorse the Barcelona Clinic Liver Cancer (BCLC) staging system as a primary prognostic model and a allocating tool of HCC treatment^2,3^.

However, there is a significant discrepancy in the initial treatment choice for HCC between the recommendations from the BCLC system and real clinical practice^4,5^. This is partially because treatment decision for HCC is highly multifactorial, in which physicians need to take into consideration the HCC stage, baseline liver function, and performance status. Moreover, other factors such as location and distribution of tumour, presence of intermediate nodule, comorbidities, socio-economic status, availability of potential living related-donors, and the invasiveness and feasibility of each treatment option play critical roles in determining the clinical outcomes of patients with HCC. Such complex nature of HCC treatment decision has hindered large-sized clinical studies, because conventional statistical methods fall short of aptly controlling multiple variables and factors.

Recent attempts on applying the artificial intelligence (AI) technique to clinical practice have focused on using AI to develop clinical decision support system (CDSS)^6–11^. For this study, we reasoned that machine learning, an application of AI that self-improves by learning from large amounts of data, would be useful for generating an algorithm for that evaluates multiple pretreatment variables to recommend optimal treatment options for HCC^12^. We believed that a good-quality database is essential, and the definition and selection of pretreatment variables are also significantly important for clinically plausible results. In this study, we thus gathered a team of well experienced hepatologists and AI scientists at our centre and developed a CDSS algorithm that can recommend optimal initial treatment for patients with HCC and predict the overall survival (OS) of patients after treatment, based on our centre’s experiences.

## Methods

### Study population

We retrospectively reviewed hospital records of 1,650 consecutive patients who were newly diagnosed with HCC at Asan Medical Center (Seoul, Korea) between January and October 2010 (Supplementary Fig. 1). Patients who had a treatment history of HCC (N = 356), those who received HCC treatment at other hospitals (N = 138), those who had a metastatic liver cancer (N = 71), those with secondary malignancies that might affect survival (N = 36), those with combined hepatocellular-cholangiocarcinoma (N = 21), and those with incidentally detected HCC after transplantation (N = 7) were excluded from the study. Consequently, the study cohort included 1,021 patients with HCC.

**Figure 1:**
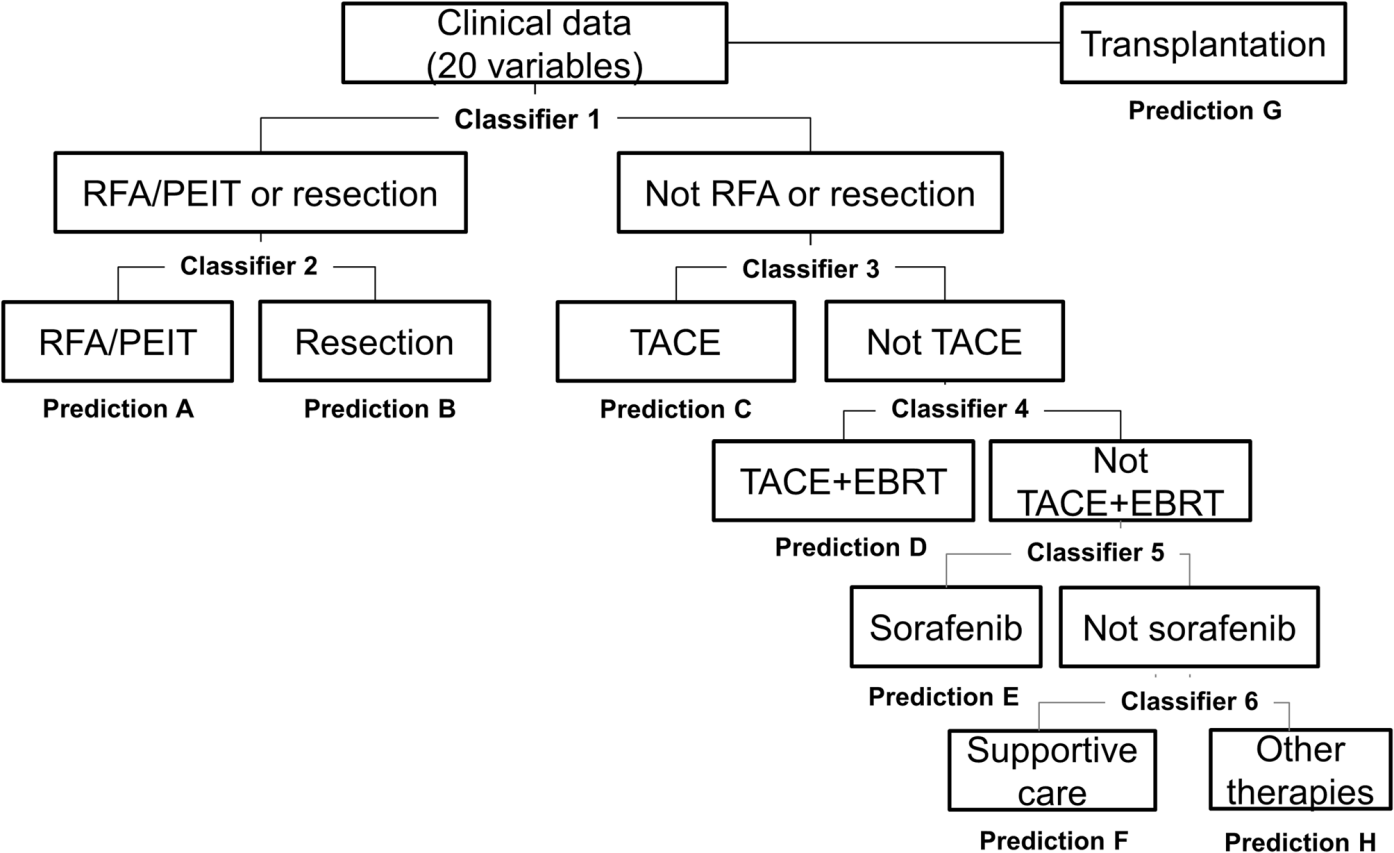
Conceptual frame of the clinical decision support system (CDSS) model for hepatocellular carcinoma. The input patient- and tumor-related variables (*N* = 20) were processed with the algorithm for treatment recommendation with multi-step classifiers in a hierarchical manner. Once the treatment option is selected, the CDSS model generates the predicted survival curve for each patient.

All enrolled patients were diagnosed with HCC using liver protocol computed tomography or magnetic resonance imaging or liver biopsy according to the current guidelines of the American Association for the Study of Liver Diseases^13^. Patients were randomly allocated to the derivation or validation set at a ratio of 4:1. The protocols of this study were approved by the Institutional Review Board of Asan Medical Center (IRB number: 2017-0188), and the requirement for informed consent from patients was waived due to the retrospective nature of the study. All methods were performed in accordance with the relevant guidelines and regulations.

### Data collection

We used our institutional database to collect information on the initial treatment option, initial treatment response, and OS of all patients. We retrospectively collected pre-treatment demographic, clinical, and imaging variables (Supplementary Table S1), treatment information and survival status of all the 1,021 patents from our centre’s database. The following demographic factors were assessed: age, sex, Eastern Cooperative Oncology Group (ECOG) score, aetiology of liver disease, presence of potential liver-related donor, body mass index (BMI), occupation, resident area, patients’ educational attainment, maximum tumour size, tumour number, tumour type (infiltrative or nodular), tumour enhancement pattern, tumour distribution, portal vein invasion, hepatic vein or inferior vena cava invasion, bile duct invasion, extrahepatic metastases, presence of dysplastic nodule, radiofrequency ablation (RFA) feasibility, presence of cirrhosis, Child–Pugh class, presence of varix, laboratory findings including alpha-feto protein (AFP) level, within or above the Milan criteria, initial treatment option, initial treatment response, and OS. RFA feasibility was defined as a size or location of the tumour to receive percutaneous RFA successfully without significant complications, evaluated by a single hepatologist, G.H.C. Tumour location adjacent to the large vessel, bile duct, hepatic hilum, liver capsule or extrahepatic organ was classified as an RFA non-feasible lesion. OS was defined as the time form date of imaging diagnosis of HCC to the date of death due to any cause.

Among the 61 initial pretreatment variables, 20 key variables (Table 1) were selected based on the importance scores calculated using the automated classifier model and the survival prediction model in the derivation set. Specifically, 14 variables were patient-related factors (age, BMI, Child–Pugh class, presence of varix, presence of ascites, ECOG score, haemoglobin level, platelet count, albumin level, prothrombin time, alanine aminotransferase [ALT] level, total bilirubin level, creatinine level, and AFP level), and six were tumour-related factors (tumour number, maximal tumour size, tumour distribution, presence of portal vein invasion, presence of metastasis, and RFA feasibility). Using these 20 variables, random forest and random survival forest methods were trained and evaluated again to recommend treatment options and to predict OS respectively in both the derivation and validation sets.

**Table 1:** Key 20 variables for hepatocellular carcinoma-clinical decision support system model.

Patient-related factors (14)	
Age	Value
Body mass index, kg/m^2^	Value
ECOG performance status score	0, 1, 2, 3, 4
Child–Pugh score	5–14
Varix	Absence / presence
Ascites	Absence / controlled uncontrolled
AFP, ng/mL	Value
Haemoglobin, g/dL	Value
Platelet count, × 10^9^/mm^3^	Value
ALT, U/L	Value
Total bilirubin, mg/dL	Value
Albumin, mg/dL	Value
Prothrombin time, INR	Value
Creatinine, mg/dL	Value
**Tumour-related factors (6)**	
Tumour number	1, 2, 3, 4 or more
Maximum tumour size, cm	Value
Distribution	Single segmental / unilobar / bilobar
Portal vein invasion	Absence / unilateral / main portal or both portal vein
Metastasis	Absence / presence
RFA feasibility*	Feasible / non-feasible

*AFP* alpha-fetoprotein, *ALT* alanine aminotransferase, *ECOG* Eastern Cooperative Oncology Group, *RFA* radiofrequency ablation.

*RFA feasibility was defined as a size or location of the tumour to receive percutaneous RFA successfully without significant complications.

Treatment options were classified as follows: transplantation, surgical resection, RFA or percutaneous ethanol injection therapy (PEIT), transarterial chemoembolisation (TACE), TACE combined with external beam radiotherapy (EBRT), sorafenib treatment, supportive care, and other therapies, which included combined therapy (e.g. surgical resection with intraoperative RFA, TACE combined with sorafenib), palliative resection, intra-arterial cytotoxic chemotherapy, clinical trials, and EBRT alone. Database review was performed by one hepatologist (G.H.C.) to avoid inter-observer bias.

### Machine learning for CDSS development in HCC

The primary outcomes were accuracies of treatment recommendation and survival prediction. The index date was defined as the date when patients underwent their first liver protocol computed tomography or magnetic resonance imaging. The follow-up period for each patient was estimated from the index date to the date of death or the last follow-up date.

Due to large differences in survival between treatments, it was difficult to train a machine learning-based model of treatment recommendation and survival prediction in an integrated way. Therefore, treatment recommendation and survival prediction models were separately designed and trained. Treatment recommendation models were hierarchically designed with six classifiers in the same manner as treatment planning in clinical practice. Supervised learning was adapted to prefer curative modalities using a classifier method. Transplantation option was not included in the treatment decision algorithm due to the medical environment of severe shortage of deceased liver donor. Although transplantation was not included in the classifier model, transplantation was suggested as an option, when it met the Millan criteria. Because factors affecting the prognosis were different for each treatment, we developed survival prediction models for each treatment. Our CDSS system operated by sequentially using treatment recommendation and survival prediction models.

To develop the treatment recommendation and survival prediction model, random forest model was employed. Random forest, which is one of the representative ensemble methods, is widely used because it is powerful and relatively lighter than other ensemble methods^14,15^. Random forest constructs a number of tree-type base models and forms an ensemble through a technique called bootstrap aggregating or bagging. As the splitting rules for random forests, Gini impurity and log-rank test were used for treatment recommendation and survival prediction models, respectively. Other possible combinations of hyperparameters of models were investigated by grid search using GridSearchCV library in Scikit-learn package.

Figure 1 shows the schematic diagram for the construction of the CDSS model for HCC. The model comprised six multi-step classifiers and seven OS prediction sub-models. The input variables (*N* = 20) were processed with the algorithm for treatment recommendation with multi-step classifiers. The CDSS model for HCC was designed to prefer curative modalities (transplantation, resection, RFA or PEIT). Once a treatment option is selected, the model demonstrates the predicted OS curve for each patient. Additionally, if another treatment option is available, our CDSS model for HCC can suggest another predicted OS curve after the alternative treatment. Therefore, the model can predict different OS curves of the same patient with different treatments, which could be helpful when clinicians made treatment decisions in actual clinical setting.

### Statistical analyses

Baseline characteristics of the patients were compared using the chi-square test for categorical variables and the Mann–Whitney U test for continuous variables. Survival distributions were compared using the Kaplan–Meier method with a log-rank test. Patients in our follow-up programme who were not confirmed deceased were recorded as censored.

In the initial phase of model development, we fitted a univariate Cox proportional hazards model to the treatment decision and survival endpoints. To select variables, we employed a two-step variable selection approach. The first step was to fit a random forest model to compute a variable importance score, and the second step was to compute a relative selection frequency based on a bootstrap resampling method^16,17^.

For the validation data sets, per-patient based analysis was performed from probability values using accuracy, sensitivity, specificity, positive predictive value, and negative predictive value for each classifier. The accuracy was defined as the percentage of correctly classified instances and calculated as follows: accuracy = (TP + TN)/(TP + TN + FP + FN), where TP, TN, FP, and FN are true positive, true negative, false positive, and false negative, respectively. Each survival prediction model was validated using bootstrapping to correct for optimistic bias. Time-dependent concordance (C)-index was used to evaluate predicted survival times, which were ranked in accordance with the observed survival times. All *P*-values were two-sided and *P* < 0.05 were considered significant. The outcome of implicit feature selection of the random forest was visualised using the Gini importance^18^. SPSS version 21 (SPSS, Inc., Chicago, IL), open-source Scikit-learn package in python version 0.19.1^19^, and random Forest SRC package in R version 3.4.1 (R Core Team, Vienna, Austria)^20^ were used for statistical analyses.

## Results

### Characteristics of the study patients

We trained our CDSS system using the derivation set (N = 813) and validated it in the validation set (N = 208). Two sets were divided by stratified random splits. The same derivation and validation sets were used for both treatment recommendation and survival prediction models. A total of 460 and 128 patients died during the median follow-up periods of 37.8 (interquartile range [IQR], 8.3–84.7) and 48.6 (IQR, 8.3–83.1) months, respectively. Patients’ baseline demographics of patients are summarised in Table 2. Of the total 1,021 patients (mean age, 56.9 years), 81.8% were male, and 77.0% had positive hepatitis B virus surface antigen. Moreover, 76.3% of patients were classified with Child–Pugh class A, and 75.1% had ECOG score of 0. Regarding tumour-related factors, 41.7% of patients had multiple tumours, and the median maximal tumour diameter was 4.0 cm (IQR 2.3–8.5). Portal vein invasion and distant metastasis were confirmed in 22.8% and 12.2% of patients, respectively. BCLC stages 0, A, B, C, and D were observed in 13.4%, 26.0%, 18.0%, 36.6%, and 6.3% of patients, respectively. As an initial treatment, transplantation was performed in 4.5%, resection in 32.9%, RFA or PEIT in 7.5%, TACE in 31.5%, TACE combined with EBRT in 6.6%, sorafenib treatment in 3.0%, supportive care in 10.1%, and other therapies in 3.8% of patients. Among the other therapies, nine patients underwent resection combined with intraoperative RFA, nine underwent palliative resection, eight underwent EBRT to liver, six underwent TACE combined with sorafenib or cytotoxic chemotherapy, and four underwent intra-arterial cytotoxic chemotherapy. Moreover, three patients were enrolled in clinical trials and underwent systemic therapy. There was no significant difference between the derivation and validation set with respect to patient-, tumour-, or treatment-related variables.

**Table 2:** Baseline characteristics of the patients, tumors, and initial treatment options.

Characteristics		All patients (*N* = 1,021)	Derivation set (*N* = 813)	Validation set (*N* = 208)
Age, year		56.9 ± 10.5	56.9 ± 10.4	57.0 ± 10.8
Gender	Male	835 (81.8)	658 (80.9)	177 (85.1)
	Female	186 (18.2)	155 (19.1)	31 (14.9)
ECOG performance status	0	767 (75.1)	615 (75.6)	152 (73.1)
	1 or 2	224 (20.9)	164 (20.2)	50 (24.0)
	3 or 4	40 (3.9)	34 (4.2)	6 (2.9)
Aetiology of liver disease	HBV	786 (77.0)	631 (77.6)	155 (74.5)
	HCV	71 (7.0)	49 (6.0)	22 (10.6)
	Others	164 (16.0)	133 (15.4)	31 (14.9)
Heavy alcohol consumption	Yes	168 (16.5)	130 (16.0)	38 (18.3)
Ascites	Present	173 (17.0)	143 (17.6)	30 (14.4)
Varices	Present	312 (30.6)	252 (30.9)	60 (28.8)
Child–Pugh class	A	779 (76.3)	620 (76.3)	159 (76.4)
	B	205 (20.1)	163 (20.1)	42 (20.2)
	C	37 (3.6)	30 (3.6)	7 (3.4)
Body mass index, kg/m^2^		24.0 (22.1–26.0)	24.0 (22.1–26.0)	24.0 (22.1–25.8)
Tumour number	1	595 (58.3)	471 (57.9)	124 (59.6)
	2–3	217 (21.2)	178 (22.9)	39 (18.7)
	≥ 4	209 (20.5)	164 (20.2)	45 (21.6)
Maximal tumour size, cm		4.0 (2.3–8.5)	4.0 (2.3–8.6)	4.0 (2.5–7.6)
Distribution	Single segmental	475 (46.5)	378 (46.5)	98 (47.1)
	Unilobar	245 (24.0)	196 (24.1)	49 (23.6)
	Bilobar	300 (29.4)	239 (29.4)	61 (29.3)
Distant metastasis	Present	125 (12.2)	99 (12.2)	26 (12.6)
Vascular invasion	Unilateral	150 (14.7)	115 (14.1)	35 (16.8)
	Main or bilateral	83 (8.1)	65 (8.0)	18 (8.7)
RFA feasibility^†^	Feasible^†^	226 (22.1)	183 (22.5)	43 (20.7)
BCLC stage	0	134 (13.1)	102 (12.5)	32 (15.4)
	A	265 (26.0)	218 (26.8)	47 (22.6)
	B	184 (18.0)	152 (18.7)	32 (15.4)
	C	374 (36.6)	287 (35.3)	87 (41.8)
	D	64 (6.3)	54 (6.6)	10 (4.8)
Laboratory findings	AFP, ng/mL	42.1 (6.7–838.2)	41.9 (7.0–827.3)	42.4 (6.9–636.1)
	Haemoglobin, g/dL	13.5 (12.2–14.6)	13.5 (12.2–14.7)	13.5 (12.2–14.5)
	Platelet count, × 10^9^/mm^3^	143 (97–197)	145 (97–197)	138 (98–199)
	ALT, U/L	37 (25–53)	37 (25–53)	39 (25–59)
	Total bilirubin, mg/dL	1.0 (0.7–1.4)	1.0 (0.7–1.4)	1.0 (0.8–1.5)
	Albumin, mg/dL	3.6 (3.2–4.0)	3.6 (3.2–4.0)	3.7 (3.2–4.0)
	Prothrombin time, INR	1.07 (1.01–1.17)	1.07 (1.01–1.17)	1.07 (1.01–1.18)
	Creatinine, mg/dL	0.8 (0.7–0.9)	0.8 (0.7–0.9)	0.8 (0.7–0.9)
Initial treatment	Transplantation	46 (4.5)	36 (4.4)	10 (4.8)
	Resection	336 (32.9)	268 (33.0)	68 (32.7)
	RFA or PEIT	77 (7.5)	61 (7.5)	16 (7.7)
	TACE	322 (31.5)	254 (31.2)	68 (32.7)
	TACE combined with EBRT	67 (6.6)	53 (6.5)	14 (6.7)
	Sorafenib treatment	31 (3.0)	24 (3.0)	7 (3.4)
	Supportive care	103 (10.1)	86 (10.6)	17 (8.2)
	Other therapies	39 (3.8)	31 (3.8)	8 (3.8)

*AFP* alpha-fetoprotein, *ALT* alanine aminotransferase, *BCLC* barcelona clinic liver cancer, *EBRT* external beam radiotherapy, *ECOG* Eastern Cooperative Oncology Group, *HBV* hepatitis B virus, *HCV* hepatitis C virus, *INR* international normalized ratio, *PEIT* percutaneous ethanol injection, *RFA* radiofrequency ablation, *TACE* transarterial chemoembolization.

*Variables are presented as mean ± standard deviation or median (IQR).

^†^RFA feasibility was defined as a size or location of the tumor to receive percutaneous RFA successfully without significant complications.

### Survival of the study patients according to the initial treatment

Supplementary Figure S2 shows the Kaplan–Meier survival curve according to the initial treatment in all patients. The 5-year survival rates of transplantation, resection, and RFA/PEIT were 86.5%, 73.7%, and 70.5%, respectively. The median survival of TACE, TACE + EBRT, sorafenib treatment, and other therapies and supportive care were 32.7 (95% confidence interval [CI] 27.0–38.4), 9.5 (95% CI 7.3–11.7), 4.2 (95% CI 2.7–5.8), 10.6 (95% CI 5.9–15.4), and 2.3 months (95% CI 1.6–3.1), respectively.

### Performance of the treatment recommendation classifier of the CDSS model for HCC

Table 3 shows the accuracy of the six classifier models trained from the derivation set. The recommended treatment from the model was compared with the treatment used in real clinical practice in the validation set. Overall, our CDSS classifier model for HCC was well generalised and showed good performance, and its standard deviations were higher in the lower branches of the treatment (e.g. sorafenib treatment, supportive care, other therapies) as the number of patients were relatively smaller. The accuracies of classifiers 1, 2, 3, 4, and 5 were 81.0% (curative treatments versus not curative treatments), 88.4% (resection versus RFA/PEIT), 76.8% (TACE vs. or not TACE), 76.6% (TACE + EBRT versus not TACE + EBRT), 80.0% (sorafenib treatment versus not sorafenib treatment), and 80.1% (supportive care versus other therapies), respectively. Supplementary Figure S3 shows the importance of the features ranked by the Gini importance that calculates reduced impurity in all trees.

**Table 3:** Accuracy, sensitivity, specificity, positive predictive value, and negative predictive value for the six classifier models in the validation set.

	Accuracy (%)	Sensitivity (%)	Specificity (%)	PPV (%)	NPV (%)
**Classifier 1** (RFA/PEIT or resection vs. not RFA/PEIT or resection)	81.0 ± 2.6	77.4 ± 4.1	83.7 ± 3.3	77.8 ± 3.6	83.5 ± 2.5
**Classifier 2** (RFA/PEIT vs. resection)	88.4 ± 3.1	56.2 ± 11.6	95.8 ± 2.7	76.8 ± 12.1	90.6 ± 2.3
**Classifier 3** (TACE vs. not TACE)	76.8 ± 2.9	82.3 ± 4.1	69.3 ± 5.5	78.3 ± 4.0	74.6 ± 4.9
**Classifier 4** (TACE + EBRT vs. not TACE + EBRT)	76.6 ± 4.7	43.9 ± 12.6	89.4 ± 3.9	61.6 ± 10.8	80.4 ± 4.3
**Classifier 5** (Sorafenib vs. Not sorafenib)	80.0 ± 4.2	12.3 ± 13.3	95.0 ± 4.0	44.0 ± 37.7	83.1 ± 3.0
**Classifier 6** (Supportive care vs. Other therapies)	80.1 ± 6.3	53.0 ± 17.6	90.4 ± 5.2	67.7 ± 15.8	83.7 ± 5.6

*EBRT* external beam radiotherapy, *NPV* negative predictive value, *PEIT* percutaneous ethanol injection therapy, *PPV* positive predictive value, *RFA* radiofrequency ablation, *TACE* transarterial chemoembolisation.

*Variables are presented as mean ± standard deviation.

### Performance of survival prediction of the CDSS model for HCC

**Figure 2:**
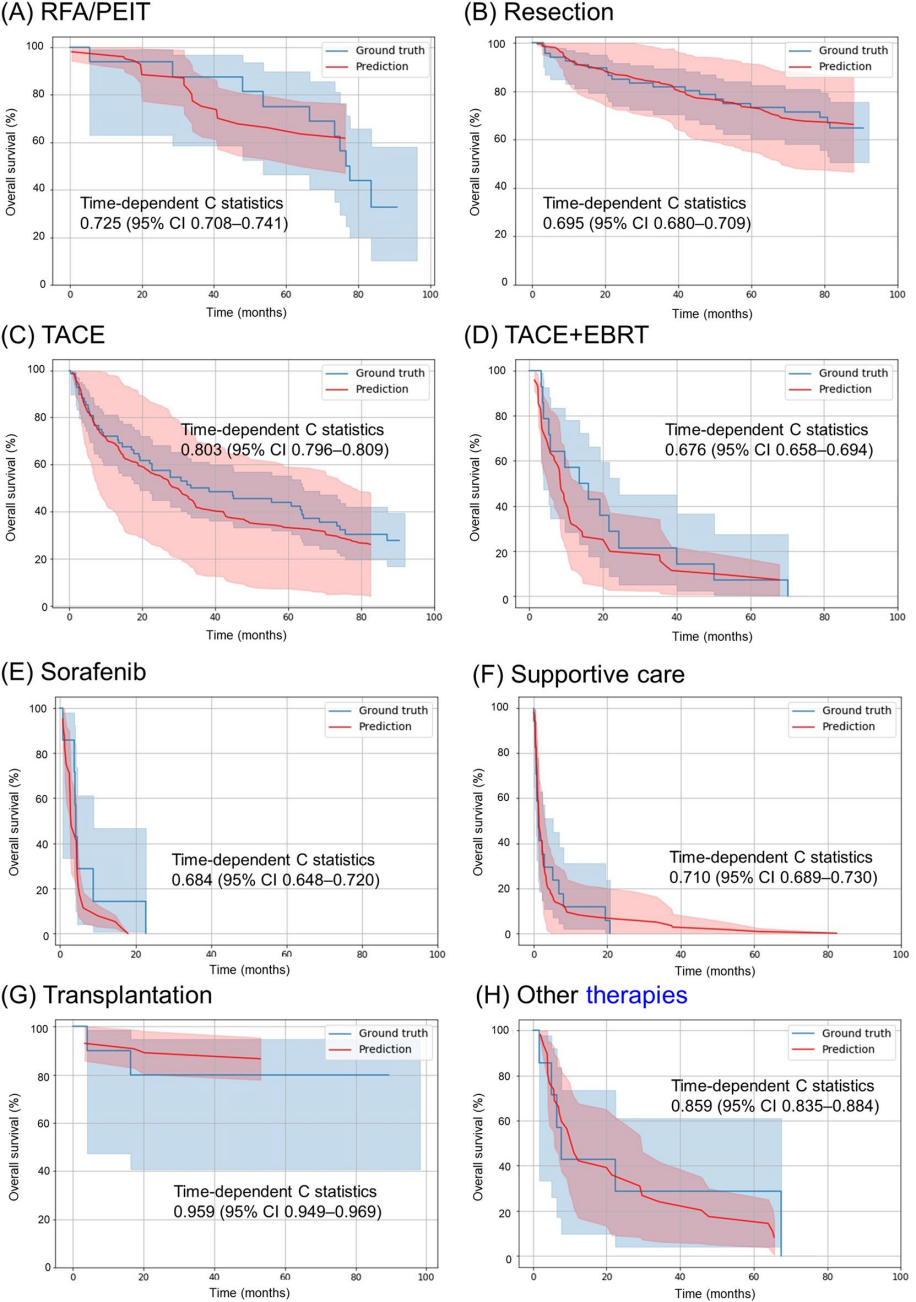
True and predicted overall survival according to the initial treatment in the validation set. (**A**) Radiofrequency ablation/percutaneous ethanol injection therapy. (**B**) Resection. (**C**) Transarterial chemoembolisation (TACE). (**D**) TACE combined with external beam radiotherapy. (**E**) Sorafenib treatment. (**F**) Supportive care. (**G**) Transplantation. (**H**) Others thepies.

Figure 2 shows predicted survival curves of each recommended treatment in the validation set. The ‘Ground truth curves’ represent the Kaplan–Meier survival curve of patients in the validation set in real clinical practice. The C-index values for the derived models of OS for RFA/PEIT, resection, TACE, TACE + EBRT, sorafenib treatment, supportive care, transplantation, and other therapies were 0.725 (95% CI, 0.708–0.741), 0.695 (95% CI, 0.680–0.709), 0.803 (95% CI, 0.796–0.809), 0.676 (95% CI, 0.658–0.694), 0.684 (95% CI, 0.648–0.720), 0.710 (95% CI, 0.689–0.730), 0.959 (95% CI, 0.949–0.969), and 0.850 (95% CI, 0.835–0.884), respectively. Supplementary Figure S4 shows the importance of the features for OS prediction in each recommended treatment.

## Discussion

In the present study, we developed a machine learning-based CDSS algorithm for recommending initial treatment option for HCC by employing clinical data from 1,021 patients. Treatment recommendations made by the CDSS model for HCC showed high accordance with the actual treatment choices, and the OS prediction was also highly associated with the observed 5-year survival rates.

**Figure 3:**
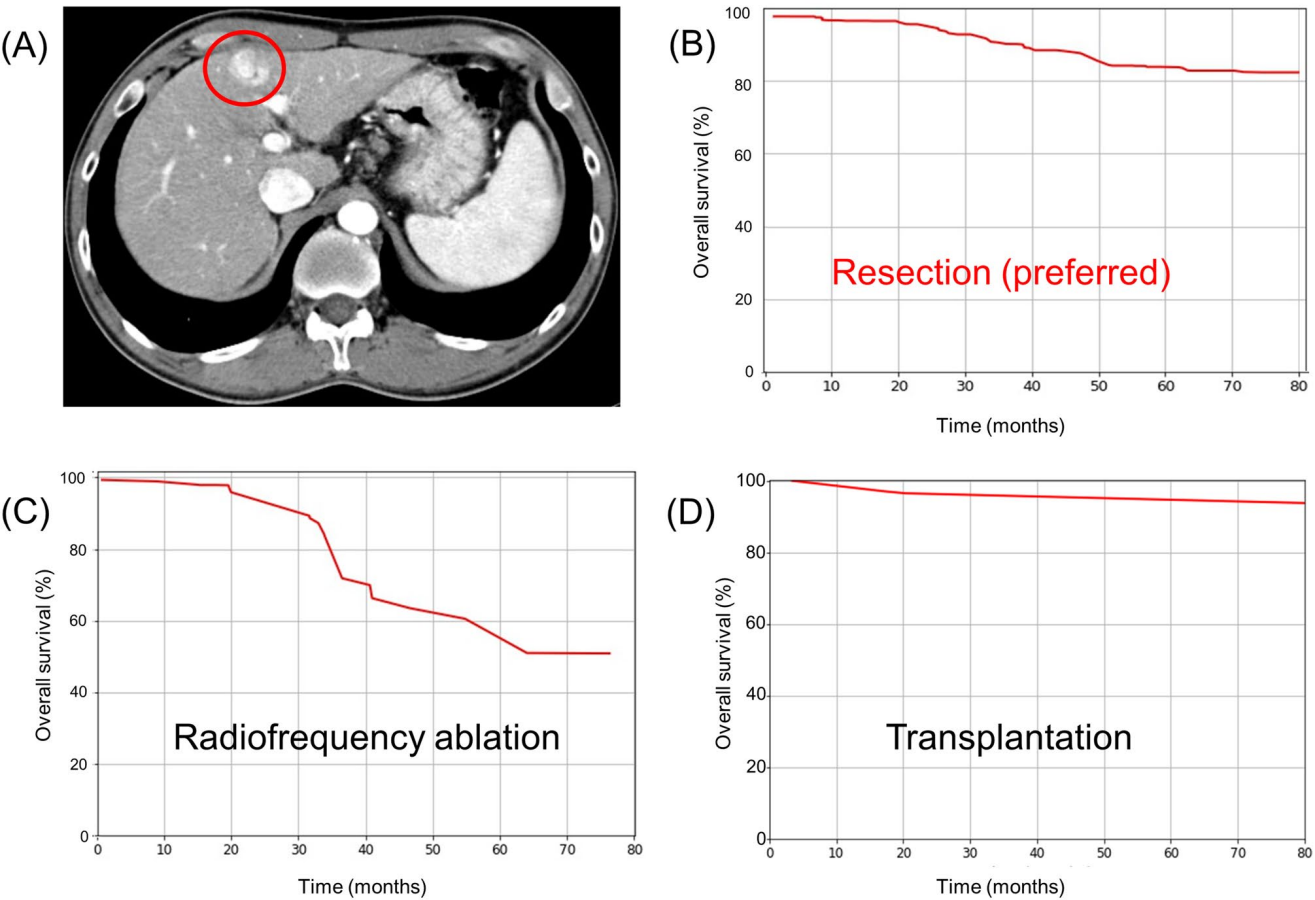
Example of application of the clinical decision support system (CDSS) model for hepatocellular carcinoma (HCC). A 43-year-old male patient with Child–Pugh class A liver function. (**A**) Arterial phase of liver protocol computed tomography showed approximately 2 cm sized single HCC. (**B**) Predicted survival curve after resection, which is the preferred option according to the CDSS model for HCC. (**C**) Predicted survival curve after radiofrequency ablation. (**D**) Predicted survival curve after transplantation.

We present a detailed example of the application of the CDSS model for HCC (Fig. 3). A 43-year-old male patient had Child–Pugh class A and a 2-cm-sized single HCC without evidence of vascular invasion and extrahepatic metastasis. The patient’s clinical details were as follows: ECOG score 0, haemoglobin 12.2 g/dL, platelet count 92 × 10^9^/mm3, albumin 3.4 g/dL, ALT 46 U/L, total bilirubin 1.0 mg/dL, creatinine 0.7 mg/dL, and AFP 42.4 ng/mL. The HCC CDSS model recommended resection as the initial treatment and the predicted 3-year and 5-year survival rates were 90.2% and 83.4%, respectively. The CDSS model for HCC also provided an estimated survival rate for RFA and transplantation, for which the predicted 5-year survival rates were 51.5% and 94.7%, respectively. In real clinical practice, this patient initially underwent resection, experienced HCC recurrence 3.2 year after the resection, received subsequent multiple on-demand TACE treatments, and still survived for a total of 6.9 years following resection.

For the development of the CDSS model for HCC, we adapted the machine learning method to overcome the complexity of treatment decision for HCC. We took special care in selecting the proper pretreatment variables and constructing high-quality database in order to ensure that the algorithm training goes well to produce applicable results. We first recruited 61 variables that are known to influence HCC treatment decision in daily clinical practice, and inputted them in a hierarchical classifier model. Through a refinement process, we finally selected 20 key pre-treatment variables with the highest importance in our model, and the resulting CDSS model proved to have good prediction ability for both treatment option and OS in the validation set.

To the best of our knowledge, this is the first description of a machine learning-based CDSS model developed for treatment decision and survival prediction in HCC. Our CDSS model for HCC not only provides the best treatment option, but also suggests alternative treatment and predicts prognosis after each treatment. Our results show that the CDSS model for HCC may be used as a supplementary system for physicians in deciding the treatment option for HCC and explaining their choice to the patients. Future multicentre studies using the HCC-CDSS model would allow for a more powerful comparison in the treatment patterns between centres and recommend treatment options with relative strength according to each centre.

Previous studies that used AI to study HCC have primarily focused on prognosis prediction after resection or TACE^6,9,10,21,22^. A recent study employed deep learning to identify multi-omics features associated with the differential survival of patients with HCC^23^. Compared to the algorithms used in previous studies, our algorithm focused more on the clinical and radiological parameters and could thus be more easily used in daily clinical practice. The integration of individual genetic information to the HCC CDSS model would enable physicians to make a more accurate selection for HCC treatment in each patient.

The Watson for Oncology, a cognitive computing system trained at the Memorial Sloan Kettering Cancer Center (New York, USA), uses natural language processing and machine learning to provide treatment recommendations. The Watson system processes structured and unstructured data from medical literature, treatment guidelines, medical records, imaging, laboratory and pathology reports, and the expertise of the physicians at Memorial Sloan Kettering to formulate therapeutic recommendations^24^. However, the Watson system has yet to be adapted for use in HCC, which may partially be due to complexity of factors that affect the treatment decision in HCC.

Our algorithm adapted manual database input in the development, and human efforts are certainly required during data acquisition. However, we already started another AI study, allowing us to automatically learn the radiological information of HCC, and training our algorithm more easily in the future learning process.

Our algorithm could not properly classify patients who received living related donor liver transplantation (LDLT) in the derivation set. As a possible explanation, it was generated in the medical environment of severe shortage of deceased liver donor. LDLT is the main method used when performing liver transplantation in our centre. Decision process of LDLT could be significantly different from that of other treatments, probably because not only availability of living donors and ethical and economic problems but also treatment willingness of the patients could influence significantly more to the decision of LDLT. Therefore, our algorithm could not recommend LDLT in the relevant patients. Hence, a different process in the decision of LDLT in patients with HCC within the Milan criteria should be considered. However, our algorithm could even predict the survival of a patient if he/she has a certain condition that requires LDLT as an initial treatment.

The present study has the following limitations. First, in this study, we trained our algorithm only for the initial treatment option and not for subsequent treatments after recurrence. Moreover, our algorithm was trained using a database from a single centre located in a HBV-endemic area with mostly male patients. Therefore, the HCC-CDSS model may show less power when used in centres with different demographics (e.g. ethnicity, aetiology, level of hospital facility, socio-economic status of the country, and even reimbursement policy), where the optimal treatment option would be different. Second, this study comprised a relatively small sample size included for each treatment specially transplantation, RFA, and sorafenib treatment. Although the c-index of the survival prediction model for these treatments was at an acceptable level, additional validation is required. Therefore, we look forward to expanding our database with the collaboration with diverse medical centres through online web-site, allowing and to make our algorithm to be more suitable for use in diverse clinical environments. Finally, although patient’s preference is one of the important factors in making treatment decisions, this variable was not included in this model. However, this cannot be quantified only by the patient's age and financial status. Therefore, we tried to compensate it by presenting the survival curve of preferred and alternative treatments.

We are more than willing to share our algorithm from the web-site with any centre worldside baseed on collaboration. This algorithm was built basically from our clinical practice and could function differently in other centres, but, hopefully, a future multicentre study could widen the usefulness of our algorithm as a method of efficacy comparison between different centres.

In conclusion, we developed HCC CDSS model for treatment decision and prognosis prediction in patients with HCC. This algorithm is considered benefical to physicians when discussing with HCC patients and when establishing a treatment decision for the appropriate initial treatment based on the estimated survival according to each treatment option, specially in HBV-endemic area. Further CDSS model with the integration of genetic information and automatically acquired imaging data could enable more individualised treatment to each patient.

## Supplementary information

Supplementary file1

## Abbreviations

AFP: Alpha-fetoprotein
AI: Artificial intelligence
ALT: Alanine aminotransferase
BCLC: Barcelona Clinic Liver Cancer
BMI: Body mass index
CDSS: Clinical decision support system
LDLT: Living related donor liver transplantation
EBRT: External beam radiotherapy
ECOG: Eastern Cooperative Oncology Group
HCC: Hepatocellular carcinoma
IQR: Interquartile range
OS: Overall survival
PEIT: Percutaneous ethanol injection therapy
RFA: Radiofrequency ablation
TACE: Transarterial chemoembolisation
