# Supplementary material for: Development of machine learning-based clinical decision support system for hepatocellular carcinoma
Source: Sci Rep. 2020 Sep 9;10:14855. doi: 10.1038/s41598-020-71796-z (PMC7481788; doi:10.1038/s41598-020-71796-z)
Supplement: Supplementary file 1 — Supplementary file1 [file 41598_2020_71796_MOESM1_ESM.doc]

**Supplementary Materials**

**Development of Machine Learning-Based Clinical Decision Support System for Hepatocellular Carcinoma**

**Gwang Hyeon Choi, Jihye Yun, Jonggi Choi, Danbi Lee, Ju Hyun Shim, Han Chu Lee, Young-Hwa Chung, Yung Sang Lee, Beomhee Park, Namkug Kim, and Kang Mo Kim**

**Supplementary Table S1. Initially assembled 61 pretreatment variables**

| **Patient variables**  **(*N* = 30)** | **Laboratory variables**  **(*N* = 13)** | **Tumour variables**  **(*N* = 18)** |
| --- | --- | --- |
| **Epidemiology**  Sex  Age  Performance status (ECOG)  Body mass index  **Aetiology**  Alcohol history  Amount of alcohol intake  Smoking history  HBsAg  HBeAg  HBeAb  HBV DNA  History of HBV Treatment  HCV Ab  HCV RNA  History of HCV treatment  **Liver cirrhosis-related**  Child-Pugh class  Varix  Ascites  Hepatic encephalopathy  Presence of splenomegaly  **Accompanying comorbidities**  Hypertension  Diabetes mellitus  Dialysis  Heart disease  Pulmonary disease  **Socio-economic status**  Marriage  Potential donor  Occupation  Education  Residence area | WBC count  Haemoglobin  Platelet count  PT (INR)  Creatinine  Estimated glomerular filtration rate  Albumin  AST  ALT  Total bilirubin  AFP  PIVKA-II  ICG test | Enhancement pattern  Tumour type  Tumour number  Maximal tumour diameter  Tumour distribution  RFA feasibility*  Presence of dysplastic nodule  Presence of portal vein invasion  Location of portal vein invasion  Presence of hepatic vein invasion  Presence of IVC invasion  Presence of bile duct invasion  Presence of metastasis  Presence of clinically significant metastasis  Location of metastasis  BCLC stage  Milan criteria  Asan criteria |

Abbreviations: AFP, alpha-fetoprotein; ALT, alanine transaminase; AST, aspartate transaminase; BCLC, Barcelona clinic liver cancer; ECOG, Eastern Cooperative Oncology Group; HBsAg, hepatitis B surface antigen; HBeAg, hepatitis B envelope antigen; HbeAb, hepatitis B envelope antibody; HBV, hepatitis B virus; HCV, hepatitis C virus; ICG, indocyanine green; INR, international normalized ratio; IVC: inferior vena cava; PIVKA-II, protein induced by vitamin K absence or antagonist II; PT, prothrombin time; RFA, radiofrequency ablation; WBC, white blood cell.

*RFA feasibility was defined as a size or location of the tumor to receive percutaneous RFA successfully without significant complication.

**Supplementary Figure S1. Patient flow gram**


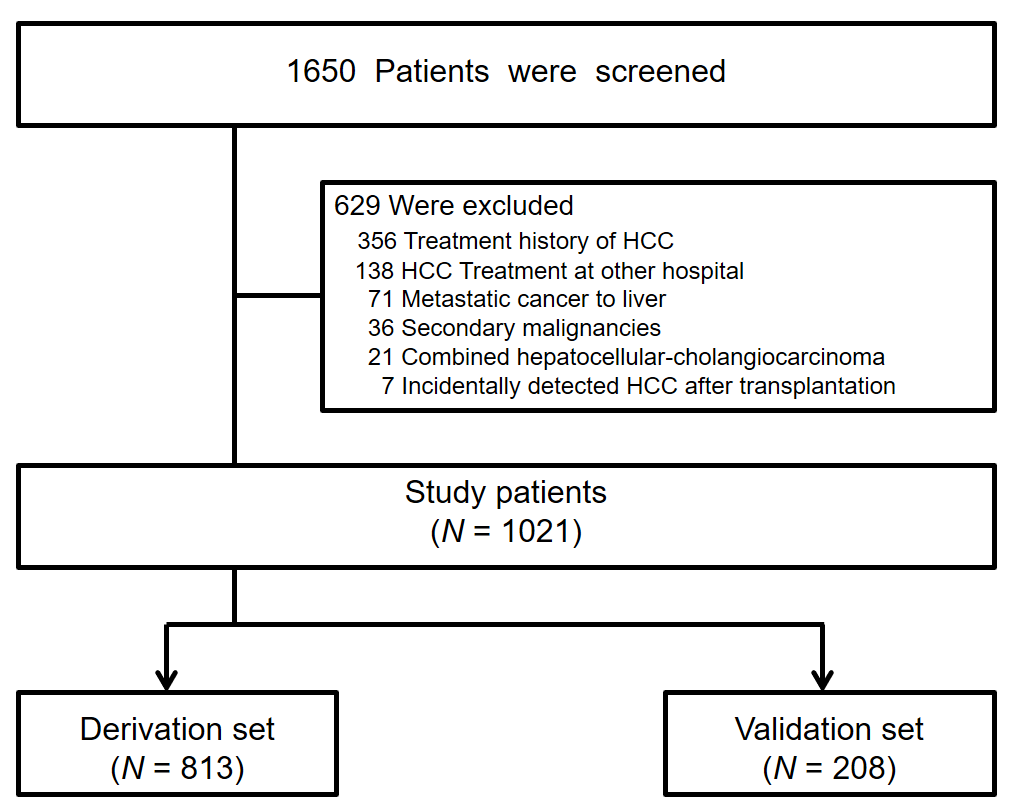


**Supplementary Figure S2. Kaplan-Meire curves according to the initial treatment in all patients.**


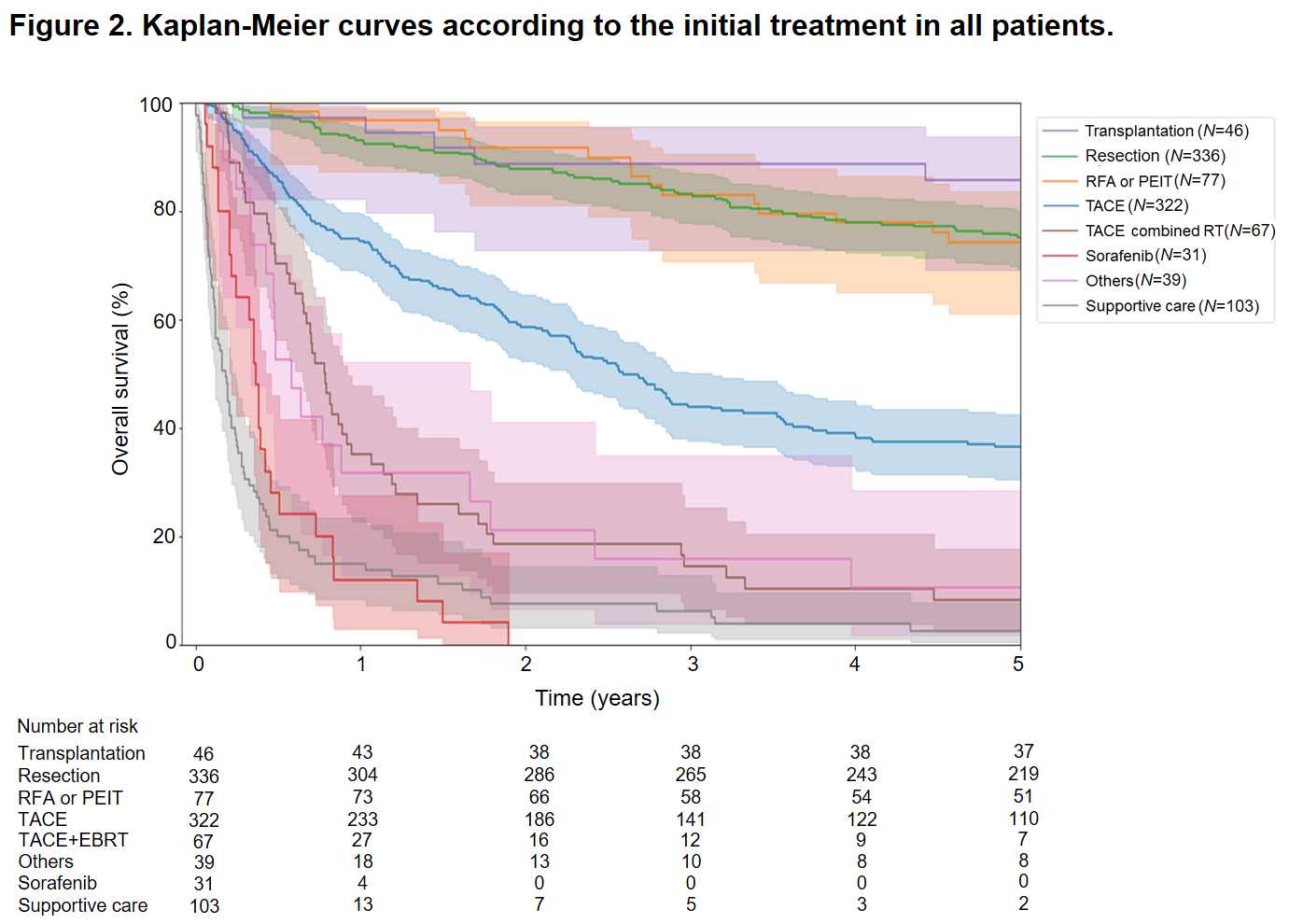


**Supplementary Figure S3. Importance of feature for 6 classifiers**

**
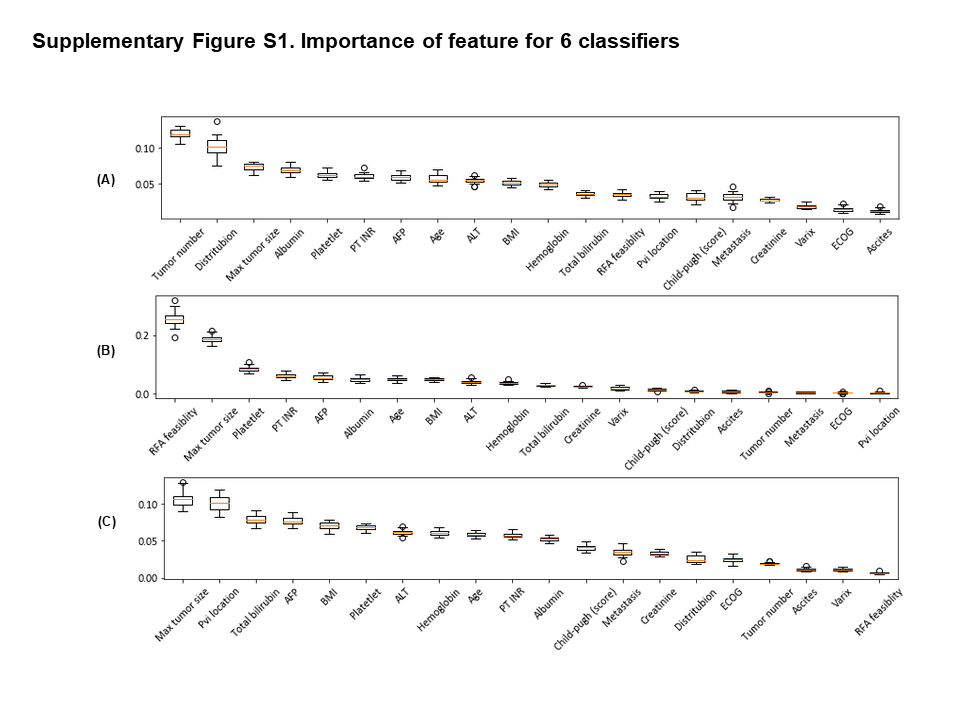

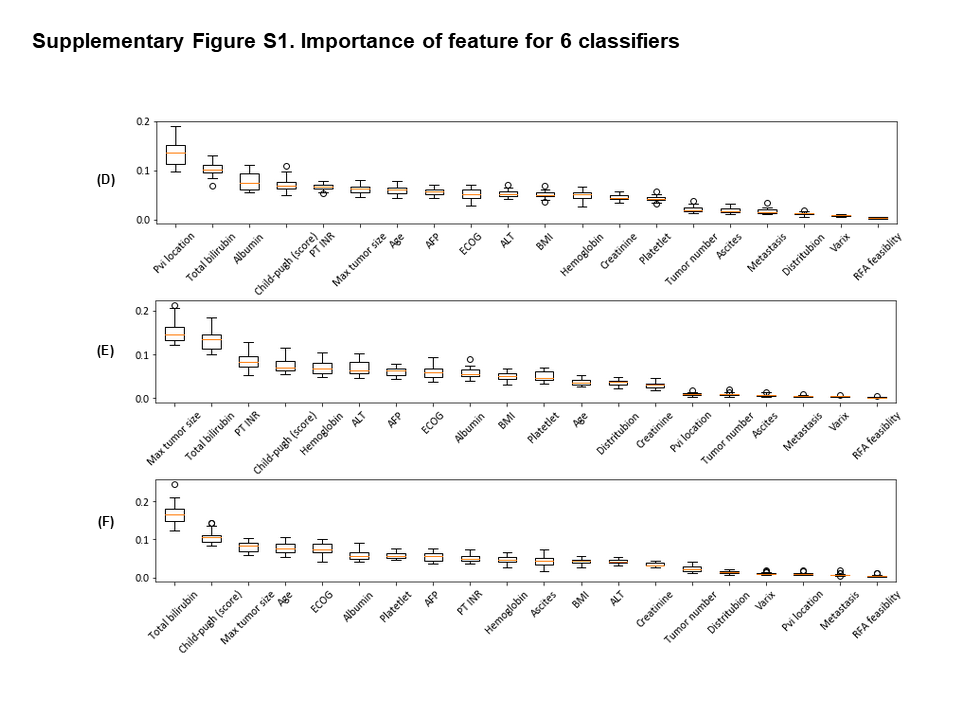
**

**Supplementary Figure S4. Importance of feature for 8 survival prediction models**

**
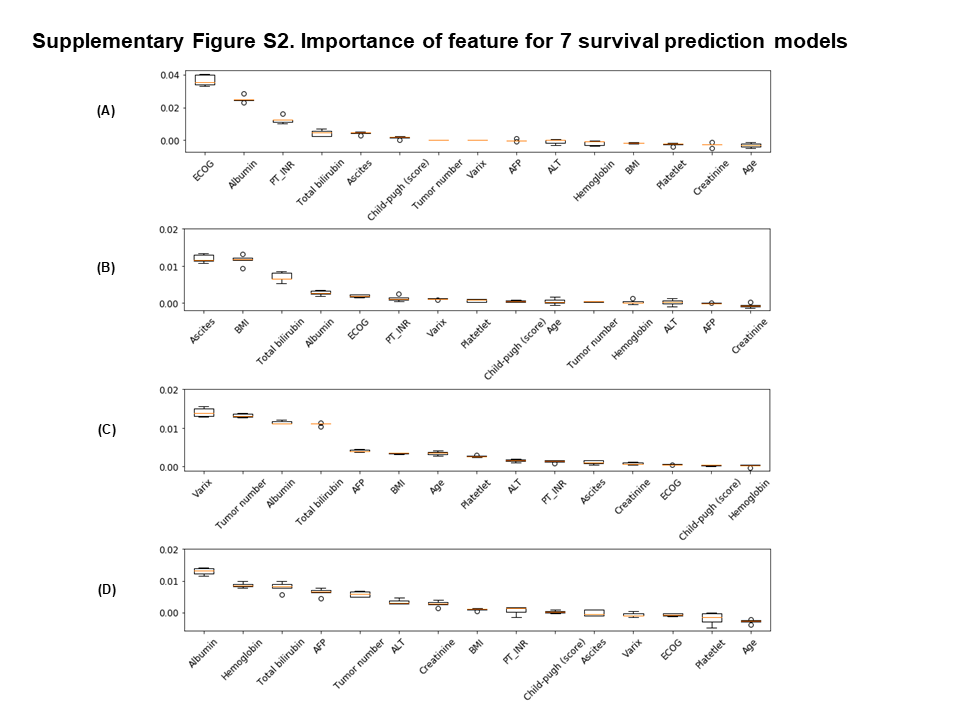

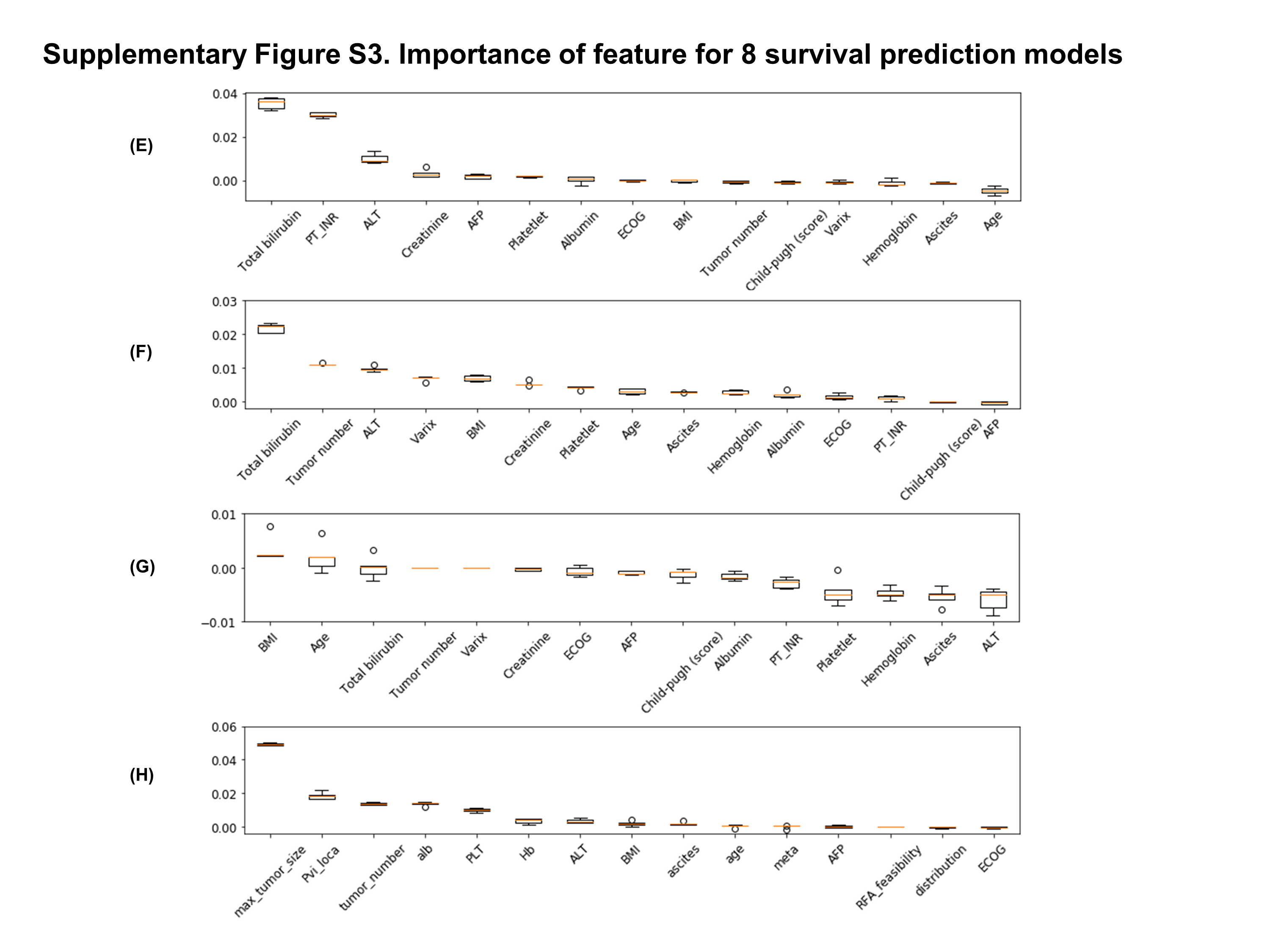
**
